# Understanding simulation-based learning for health professions students from culturally and linguistically diverse backgrounds: a scoping review

**DOI:** 10.1007/s10459-024-10384-6

**Published:** 2024-11-07

**Authors:** Luocheng Zhang, Freyr Patterson, Adriana Penman, Roma Forbes

**Affiliations:** School of Health and Rehabilitation Sciences, The University of Queensland, St Lucia, Brisbane 4067 Australia

**Keywords:** Scoping review, Culturally and linguistically diverse, Health professions, Education, Simulation, Participation

## Abstract

Simulation-based learning (SBL) is an important component in health professions education and serves as effective preparation or a substitution for clinical placements. Despite their widely accepted benefits, students from culturally and linguistically diverse (CALD) backgrounds may not experience the same learning outcomes from engaging in SBL as their local peers due to complex factors. Supporting students from CALD backgrounds in SBL is vital, not only to optimise their learning experiences and outcomes, but also ensure inclusive health professions education. While the literature on the participation of students from CALD backgrounds in SBL activities is emerging, this scoping review was conducted to (1) map the evidence on how SBL impacts the learning outcomes of health professions students from CALD backgrounds; and (2) understand how students from CALD backgrounds perceive their SBL experiences. Following Arskey and O’Malley’s framework and Joanna Briggs Institute methodology for scoping reviews, a search was conducted in January 2024 using PubMed, Embase, CINAHL, Scopus, PsycINFO, and ERIC. Ten papers met the inclusion criteria. This review highlighted three themes: (1) diverse learning outcomes of SBL; (2) facing linguistic and cultural challenges that are inherent to SBL; and (3) preparation, reflection, and support to actively participate in SBL activities. This review indicates that SBL could enhance clinical skills and confidence in students from CALD backgrounds. However, well-designed SBL activities to meet the learning needs of students from CALD backgrounds are currently lacking and further research across broader health professions fields is needed.

## Introduction

A global shortage of healthcare workers has led to a rise in health professions programs attracting a diverse student body (World Health Organisation, [WHO], 2018). Due to the internationalisation of healthcare education, high-income countries, particularly, have seen a notable rise in students from culturally and linguistically diverse (CALD) backgrounds enrolling in health professions programs (Davey, 2023). However, it should be noted that there is a significant challenge in defining CALD status and there is a lack of a universally accepted definition (Pham et al., 2021). Emerging alongside global migration, “CALD” is recognised under various terminologies in many countries such as “multicultural”, “ethnically diverse”, “ethnic minorities” and “migrant communities” (Office for National Statistics, 2021; Statistics Canada, 2016).

Within health professions education, simulation-based learning (SBL) is widely used to enhance experiential learning, and has become a critical component of many health professions programs (Loutet et al., 2020). SBL has been defined as an engaging, hands-on learning process that replicates real-world events or series of scenarios to address a problem (Dai & Ke, 2022). SBL can create supportive and safe learning opportunities for complex clinical environments or specific patient cases, where exposure might be limited during clinical placements because of the prioritisation of patient care (Lateef, 2010; Penman et al., 2021). The effectiveness and benefits of using SBL, either in preparation or as a substitute for clinical placement in health professions education, have been extensively examined in randomised controlled studies across different health professions programs (Hill et al., 2021; Imms et al., 2018; Ketterer et al., 2020; Pauli & Hughesdon, 2024; Watson et al., 2012). As such, SBL forms an integral part of the educational experience for students from CALD backgrounds.

Students from CALD backgrounds bring myriad benefits to their university community including different health practices, beliefs, and perspectives rooted in their diverse cultural backgrounds (Gilligan & Outram, 2012). Such contributions enhance campus environment diversity and enrich classroom discussions (Baker et al., 2024). Additionally, students from CALD backgrounds can bring attention to health disparities and social determinants that are specific to certain cultural or linguistic communities, fostering a more inclusive approach to local healthcare systems (Jackson & Gracia, 2014).

However, despite these benefits, students from CALD backgrounds often face additional learning needs and challenges in health professions programs. Principal challenges for students from CALD backgrounds highlighted in current literature include language barriers (Hari et al., 2021; Lin et al., 2021), struggles in adapting to unfamiliar educational settings, understanding local cultures and healthcare systems, and facing question-asking difficulty (Lim et al., 2016). Those students may also experience isolation, and in some cases, rejection, and discrimination, which would in turn limit their uptake of support and resources (Jeong et al., 2011; Lim et al., 2016).

These challenges become particularly evident in the clinical learning environment, which includes clinical placements, internships, or fieldwork - core components of entry-level health professions programs (O’Reilly & Milner, 2015). Previous research conducted in English-speaking countries has shown that students from CALD backgrounds experience a higher rate of placement failure than domestic students, with communication competence being one of the contributing factors (Attrill et al., 2012; Nicola-Richmond et al., 2017). Clinical educators also report additional challenges when working with students from CALD backgrounds (Abu-Arab & Parry, 2015). Commonly reported challenges include additional time and workload needed to supervise students from CALD backgrounds, and insufficient knowledge and resources to navigate mentoring relationships with such students (Attrill et al., 2015; Jeong et al., 2011; Hagqvist et al., 2020; O’Reilly & Milner, 2015;).

Considering the additional challenges that students from CALD backgrounds face in both classroom and clinical settings, along with the increasing recognition of the benefits of SBL in health professions education, SBL has the potential to play a critical role in supporting these students. Literature on other forms of interactive learning approaches, such as case-based learning (CBL) and problem-based learning (PBL), which share similarities with SBL, has demonstrated positive effects on students from CALD backgrounds, including increased learning motivation, active participation, and enhanced problem-solving skills through case analysis and collaboration with peers (Yang et al., 2024). However, since these student-centred pedagogical approaches rely heavily on social interactions (Gilligan & Outram, 2012; Loutet et al., 2020), students from didactic teaching backgrounds may face difficulties adapting to these participative learning methods (Jeong et al., 2011). Previous research has revealed that interactional challenges for Asian students in PBL are not solely due to language proficiency but are also influenced by different interactional behaviours, such as listening habits, cultural norms around participation, and the preference for silence versus active verbal contributions (Remedios et al., 2008). PBL research has evolved to explore not only knowledge acquisition but also social interactions in PBL, which highlights the importance of understanding the lived experiences and social dimensions of learning within PBL (Imafuku & Bridges, 2016). Similarly, SBL requires active engagement and social interactions, offering both opportunities and challenges for students from CALD backgrounds. Therefore, examining the lived learning experiences of students from CALD backgrounds in SBL, including factors that promote or hinder their learning, would provide valuable insights into how SBL can be optimally designed to meet their learning needs.

Despite the growing body of literature on the experiences of students from CALD backgrounds in health professions education (Lewis & Bell, 2020; Mikkonen et al., 2016), no review to date has explored how students from CALD backgrounds experience and navigate SBL environments. This scoping review seeks to address this gap with the aim to systematically map the evidence on how SBL impacts the learning outcomes of health professions students from CALD backgrounds and how students from CALD backgrounds perceive their SBL experiences. This is critical for further practice and research, not only to develop more inclusive and effective health professions education strategies that cater to the needs of diverse student populations, but also to maximise the benefits that students from CALD backgrounds bring, thereby enriching the learning environment for all students.

## Methods

Scoping reviews are commonly employed where the available body of literature is constrained, yet where there is a growing body of emerging evidence related to a specific subject (Levac et al., 2010). Unlike a systematic review, which focuses on synthesising research findings for a narrowly defined question with ample empirical data, a scoping review is intended to explore broader concepts or characteristics related to a research topic (Grant & Booth, 2009). Given the preliminary observation that limited literature exists on the experiences of health professions students from CALD backgrounds in SBL, a scoping review was deemed the most suitable methodology to map out the breadth and scope of the existing literature (Arksey & O’Malley, 2005; Munn et al., 2018).

This scoping review followed Arksey and O’Malley’s five-step framework (Arksey & O’Malley, 2005), which has been subsequently updated and further developed in Joanna Briggs Institute (JBI) scoping review methodology (Peters et al., 2020). Following the JBI guideline, a priori protocol was developed, agreed upon by all authors, and registered on the Open Science Framework.

### Step 1. Identify the research question

This scoping review aimed to answer the overarching research question: What is known about the impacts of SBL on health professions students from CALD backgrounds, and their perceptions of participating in SBL activities? This question was constructed according to the PCC format, in which the Population (health professions students from CALD backgrounds), Concept (impacts of SBL and the perceptions of these students regarding their participation in SBL), and Context (educational settings where SBL takes place) were described (Peters et al., 2020). Consequently, the sub-question was also addressed: What are the factors that promote or hinder the learning outcomes of health professions students from CALD backgrounds participating in SBL activities?

### Step 2. Identifying relevant studies

Articles were searched across the following databases: PubMed, Embase, CINAHL, Scopus, PsycINFO and ERIC, using search terms alongside Boolean operators (AND/OR). Key concepts were initially derived from the research question by the entire research team, which was developed based on the established PCC framework, focusing on terms associated with health professions students, simulation, and CALD. In consultation with the University’s librarian from the Faculty of Health and Behavioural Science, the original search terms (see Table 1) were used to generate suggested mesh headings and preferred search terms across different databases. This scoping review acknowledged the complexity of the CALD concept (Pham et al., 2021) and thus adopted an inclusive approach to incorporate terminology used globally that might serve in place of CALD. Using a combination of these terms and subject headings, searches were conducted within keywords, titles, and abstracts.

**Table 1:** Search strategy

Concepts	Search terms
Simulation	Simulat* OR “Simulation Training” OR “Problem-Based Learning” OR “Role Playing” OR “Manikins” OR “Role play*” OR “Role-play*” OR “Standardised patient*” OR “standardized patient*” OR “SP” OR “Case stud*” OR “problem-based” OR “problem based” OR “PBL” OR “Scenario-based” OR “Scenario based” OR Mannequin* OR mannikin* OR “Virtual Reality” OR “VR”
Student	Student*
CALD	“Cultural Diversity” OR CALD OR “culturally and linguistically diverse” OR “Cultural and linguistic diversity” OR “non English” OR “non-english” OR “English as a second language” OR ESLOR EALD OR EAL OR “English as an additional language” OR “cultural diversit*” OR “culturally diverse” OR “Language Diversit*” OR “linguistic diversit*” OR “linguistically diverse*” OR “Overseas student*” OR “international student*” OR “Foreign student*” OR “Ethnical diversit*” OR “ethnic minorit*” OR “Ethnically diverse” OR “ethnocultural diversit*” OR multicultural OR “Black Asian and Minority Ethnic” OR “BAME” OR migrant* OR refugee* OR immigrant*

### Stage 3. Study selection

Articles included in the scoping review must meet the inclusion criteria based on the PCC framework. This review encompassed studies involving students enrolled in health professions programs, with at least one primary research aim focusing on students from CALD backgrounds participating in SBL activities. Studies incorporating low to high-fidelity SBL for learning purposes were included. The review was confined to empirical research studies to enhance the reliability and validity of the synthesised findings. To obtain a comprehensive result and to ensure the historical context of the literature was captured, no date restrictions were imposed. Given the linguistic capabilities of the research team, the review was limited to studies published in English. Studies were excluded if they focused on participants who had only completed a segment of their health professions program in a country different from where their primary study was hosted (e.g., international study tours, exchanges, or placements), or if the research used case studies and problem-based learning activities not employed for learning (e.g., OSCE assessments).

A total of 1,810 references were identified from the initial database search, imported into EndNote 20 (a reference management software), and then exported into Covidence (an online platform used to manage screening of articles), where 421 duplicates were removed by the Covidence system along with one additional duplicate removed manually, and 1,388 articles remained.

In the first screening round, all titles and abstracts of the retrieved papers were reviewed by the primary investigator (L.Z.), and the remaining three research members (A.P., F.P. and R.F.) completed the second independent screening. During this screening, the research team identified that many of the articles incorporated simulation solely for assessment or examination purposes. It was then decided by the team that the review would focus on articles implementing simulation for at least one learning purpose, leading to the addition of this criterion to the above final exclusion criteria. A total of 51 papers were then deemed eligible and underwent full-text examination. The full text of one article was not able to be obtained, despite following the protocol and contacting the corresponding author but receiving no responses, and consulting the librarian yielded no further results as no full text was available. After the full-text review by two authors, 11 articles met the inclusion criteria. However, two of these articles were later excluded during the data charting stage by the research team. One article pertained to graduates from CALD backgrounds and the other concerned school children from CALD backgrounds, resulting in nine articles. One additional paper was identified and deemed relevant through the reference list check of the nine articles. A final set of 10 articles were included in the review.

**Fig. 1:**
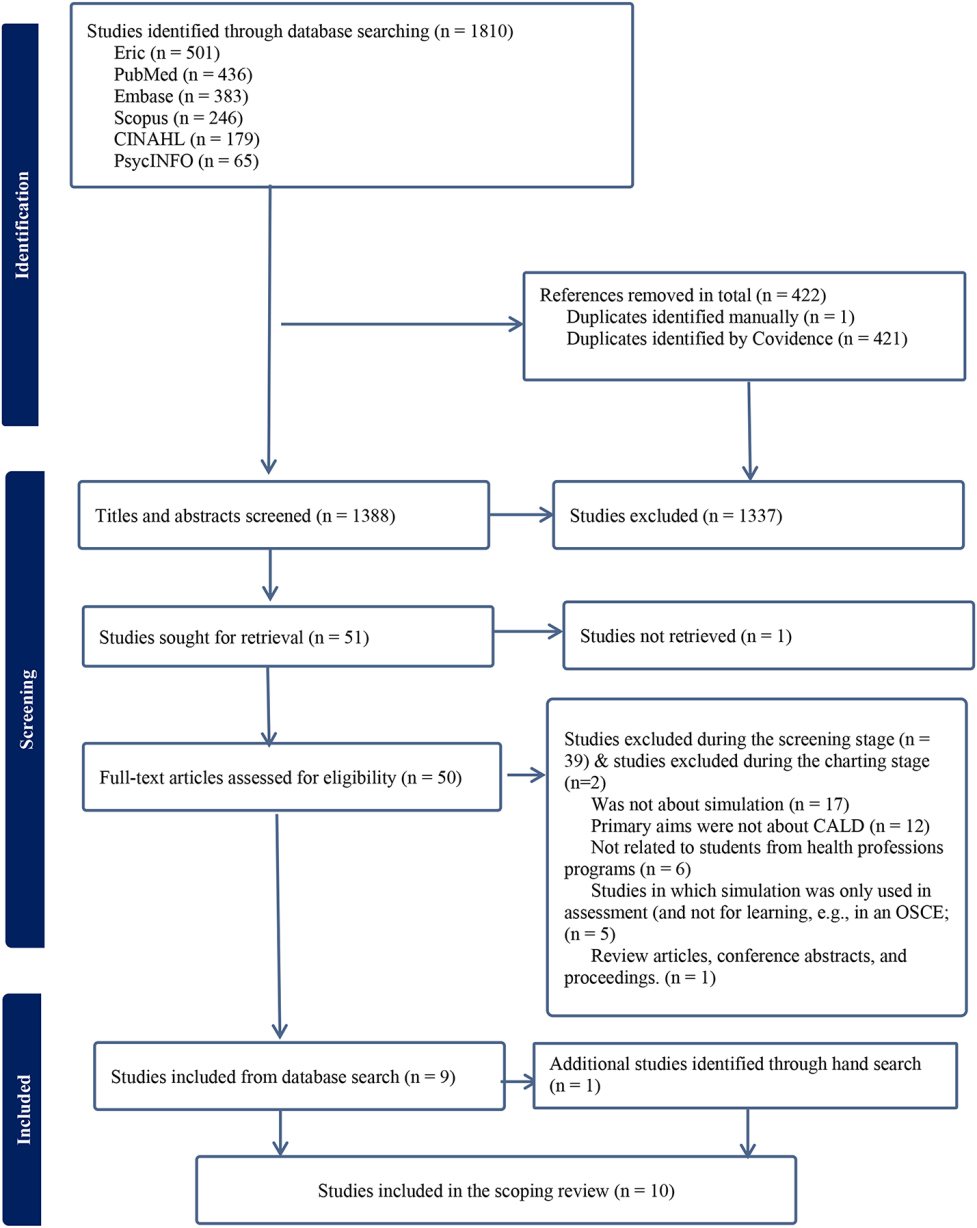
PRISMA flow diagram

The Preferred Reporting Items for Systematic Reviews and Meta-Analyses Extension for Scoping Reviews (PRISMA-ScR) (Tricco et al., 2018) flowchart was used to report the search decision process (see Fig. 1).

### Stage 4. Charting the data

A template data charting Excel table was developed by the principal researcher L.Z., structuring the potentially charted items around the PCC framework, as well as the purpose and rationale for conducting the review (Pollock et al., 2023). This was followed by a team meeting where the items on the charting table were confirmed. As a descriptive-analytical method advised by Arksey and O’Malley (2005), each article was summarised with details on journal, author(s), country of origin, year of publication, study aim, study methodology, sample details, simulation details, outcomes measured and data collection methods and key findings.

The primary investigator (L.Z.) completed the charting of all studies, while the second round of charting for each study was simultaneously conducted by the remaining team members (A.P., F.P. and R.F.). Any discrepancies identified between the chartings were resolved through discussions among all team members to ensure consistency and accuracy in the data.

### Stage 5. Collating, summarising, and reporting the results

Basic descriptive analyses were used for study demographics and quantitative data. For qualitative data, thematic analysis was employed following Braun & Clarke’s reflexive approach (2022), involving the initial coding of direct quotes or paraphrased content to initially develop broad themes. These themes were then contextualised with quantitative data to provide a more comprehensive understanding of the research findings. Final themes were generated and refined through in-depth discussions during regular team meetings. Codes and themes were independently checked and verified by another researcher (R.F.) to ensure credibility and trustworthiness. The research team consisted of a principal researcher from a CALD background with past personal experience in occupational therapy education in Australia, and other members with extensive experience within allied health, contributing pedagogical insights and expertise in SBL learning. Reflexivity was maintained throughout the analysis by regularly reflecting on the researchers’ positionality and its influence on theme development. The team engaged in iterative coding discussions to ensure rigor and depth, focusing on credibility rather than consistency and reliability in a positivist sense. The following results provide a cross-study synthesis of the data analysis.

## Results

### Study characteristics

Out of the 10 articles included, three were published before 2014, three between 2014 and 2020, and four from 2020 onwards (see Table 2). Papers were published across seven different journals with half (*n* = 5) of the papers in either BMC Medical Education (*n* = 3) or Clinical Simulation in Nursing (*n* = 2). Studies were conducted in the United States (*n* = 4), Australia (*n* = 2), and in Canada, China, Germany, and the United Kingdom (each *n* = 1).

A range of research methodologies were adopted across the studies included. Most used qualitative approaches (*n* = 5), while three employed quantitative approaches (Nagy et al., 2021; Rutledge et al., 2022; Zhang et al., 2019), and two conducted mixed-methods studies that integrated both quantitative and qualitative data (Harvey et al., 2013; Rossiter et al., 2023).

**Table 2:** Study characteristics

Author (s)	Year of publication	Journal	Country of origin	Study methodology
Adedokun et al.	2022	Journal of Professional Nursing	United States	Qualitative phenomenological study
Hussin	2013	English for Specific Purposes	Australia	Qualitative case study
Rossiter et al.	2023	BMC Medical Education	United Kingdom	Mixed methods
Rutledge et al.	2022	Journal of Obstetrics and Gynaecology Research	United States	Quantitative longitudinal study
Harvey et al.	2013	Journal of Nursing Education	Australia	Mixed-methods
Slingsby et al.	2010	International Medical Journal of Experimental and Clinical Research	United States	Qualitative phenomenological study
Zhang et al.	2019	BMC Medical Education	China	Quantitative quasi-experimental study
King et al.	2017	Clinical Simulation in Nursing	Canada	Qualitative exploratory study
Nagy et al.	2021	BMC Medical Education	Germany	Quantitative quasi-experimental study
Graham & Atz	2015	Clinical Simulation in Nursing	United States	Qualitative phenomenological study

### Participant characteristics

The health professions represented by participants across the reviewed studies were predominantly nursing (*n* = 4) and medicine (*n* = 4), with one in pharmacy (Hussin, 2013) and one interdisciplinary study that included physiotherapy, podiatry, and occupational therapy students (Rossiter et al., 2023) (see Table 3). All participants in these studies were at the undergraduate level.

Eight studies included a sample of participants from a range of cultural and ethnic backgrounds, while one study by Harvey et al. (2013) utilised the term ‘CALD’ without specifying ethnicities, and one study by Slingsby et al. (2010) focused solely on Japanese students in the United States. Three studies (Nagy et al., 2021; Rossiter et al., 2023; Rutledge et al., 2022) included a mix of students from CALD backgrounds and domestic students. 

**Table 3:** Participant characteristics

Author (year)	Health professions of participants	Educational level of participants	Sample size and cultural and linguistic backgrounds of participants
Adedokun et al. (2022)	Nursing	Undergraduate	*N* = 9, participants were from different countries, speaking various languages such as Oromo, Twi, Igbo, Yoruba, Urhobo, and Korean.
Hussin (2013)	Pharmacy	Undergraduate	*N* = 20, international students were from Malaysia with Mandarin or Cantonese as their first language.
Rossiter et al. (2023)	Physiotherapy, Podiatry and Occupational Therapy	Undergraduate	*N* = 53, including both international and domestic students. Most international students were from Asia.
Rutledge et al. (2022)	Medicine (Osteopathic Medicine)	Undergraduate	*N* = 382 including Caucasian, Asian, Latino, and others (African American, Native-American, Pacific Islander)
Harvey et al. (2013)	Nursing	Undergraduate	*N* = 122, students were from CALD backgrounds, but backgrounds were not specified.
Slingsby et al. (2010)	Medicine	Undergraduate	*N* = 10, students were from two Japanese medical schools
Zhang et al. (2019)	Medicine (Obstetrics and Gynaecology)	Undergraduate	*N* = 94, most of the participants were from Southeast Asia, particularly India.
King et al. (2017)	Nursing	Undergraduate	*N* = 35, Participants spoke various native languages including Arabic, Tagalog, Malayalam, Bengali, Afrikaans, etc., with the majority speaking Arabic.
Nagy et al. (2021)	Medicine	Undergraduate	*N* = 50, including international (*n* = 15) and local students (*n* = 28). International students came from various countries, including Syria, Kuwait, Cyprus, Cameroon, Singapore, USA, Portugal, Romania, Bulgaria, Burundi, and Peru.
Graham & Atz (2015)	Nursing	Undergraduate	*N* = 16. Most of the participants were black or African American.

### Characteristics of SBL

A range of different modes of SBL were reported in the included studies. As outlined in Table 4, most studies (*n* = 8) specified the inclusion of standardised or simulated patients as the primary method of SBL, with two studies incorporating manikins (Nagy et al., 2021; Rutledge et al., 2022) and one study utilising an online simulation environment (Rossiter et al., 2023). Furthermore, one study reported general SBL experiences as a teaching-learning strategy in nursing programs (Adedokun et al., 2022), and one used high-fidelity SBL without further specification of the simulation methods (Graham & Atz, 2015).

SBL activities predominantly took place in university-based settings, except for one which was conducted in a teaching hospital setting (Zhang et al., 2019). While three studies did not specify the purposes of simulation (Adedokun et al., 2022; Graham & Atz, 2015; Hussin, 2013), the aim of SBL in most studies (*n* = 7) was to develop participants’ clinical skills, ranging from basic communication and history-taking to procedural skills including physical examinations and specific clinical procedures. SBL in two of these studies specifically aimed to prepare participants for clinical placements (Harvey et al., 2013; Rossiter et al., 2023).

Details regarding the implementation of SBL varied; two studies included simulation as part of intensive workshops (Rossiter et al., 2023; Slingsby et al., 2010) and one study integrated SBL intermittently throughout the year as an extracurricular program (Harvey et al., 2013). Additionally, SBL in the remaining seven studies was described as embedded in the health professions programs without further details such as duration or specific steps of implementation. 

**Table 4:** Characteristics of SBL

Author (year)	Types of simulation	Settings of simulation implemented	Purposes of simulation	Simulation implementation details
Adedokun et al. (2022)	General simulation experiences	University-based	A teaching-learning strategy	- No further details were provided.
Hussin (2013)	Simulated patients	University-based	A teaching-learning strategy	- Third-year pharmacy students played the role of pharmacists while teaching staff played the role of patients through weekly scenario-based dispensing and counselling simulation. - The duration was not specified.
Rossiter et al. (2023)	In-person simulation program with simulated patients; online program with interactive materials including 360° images of three different placement settings.	University-based and online learning environment.	To improve students’ preparedness and foundational skill development for clinical placements.	- The in-person simulation program included a week-long workshop with orientation, interprofessional discussions, turn-taking, and debriefing. - The online simulation program consisted of interactive materials and case study questions. - Duration of each type of simulation was not specified.
Rutledge et al. (2022)	Anatomical models and specialised standardised patients	University-based	To enhance medical students’ confidence and competency with performing a female genitourinary exam (FGUE)	- First-year medical students practiced the female genitourinary exam on anatomical models. - second-year students practiced on standardised patients, having previously used models in their first year. - The duration was not specified.
Harvey et al. (2013)	Simulated patients	University-based	To address challenges that may hinder the accomplishments of nursing students from CALD backgrounds in clinical practice.	- Four-week of 90 min role-play sessions were included in an 18-hour extracurricular program named Preparation for Practice Program running over 10 weeks in the semester.
Slingsby et al. (2010)	Simulated patients	University-based	Students to obtain case history, perform physical examination and formulate a management plan.	- Simulation was included in a 5-day workshop. - The duration of simulation was not specified.
Zhang et al. (2019)	Standardised patients	University teaching hospital	To improve the foreign medical students’ communication skills and their competency in history-taking within a safe learning environment.	- Practiced skills with the gynaecological standardised patients who were played by attending doctors. - The duration was not specified.
King et al. (2017)	Standardised patients	University-based	To create contextually rich leaning environment for nursing students from CALD backgrounds and enhance their academic success.	- Students engaged with simulated patients as part of their nursing courses which included foundational nursing, family nursing, health assessment, and mental health nursing. - The duration was not specified.
Nagy et al. (2021)	Arm prosthesis model and standardised patients	University-based	To enhance medical students’ communication skills in performing clinical procedural skills.	- Interactive role-plays were used. - The duration was not specified.
Graham & Atz (2015)	High-fidelity simulation	University-based	A teaching-learning strategy	- Participants previously had high-fidelity simulation experiences. - No further details were provided.

### Evaluation of the SBL experience

As outlined in Table 5, all the studies aimed to evaluate the impacts of SBL on the learning outcomes of students from CALD backgrounds. Most of these studies (*n* = 9) utilised self-reported outcomes to measure students’ clinical skills and confidence in different aspects. Specifically, two studies employed pre- and post-surveys with a 5-point Likert scale (Rossiter et al., 2023; Rutledge et al., 2022), one used a survey incorporating both a 5-point Likert scale statement and open-ended questions (Harvey et al., 2013), and one used a questionnaire survey (Zhang et al., 2019). Additionally, three studies included semi-structured interviews (Adedokun et al., 2022; Hussin, 2013; Slingsby et al., 2010), and four employed focus groups (Graham & Atz, 2015; Hussin, 2013; King et al., 2017; Rossiter et al., 2023). Two studies combined these methods (Hussin, 2013; Rossiter et al., 2023). Furthermore, four studies integrated the evaluation completed by others. Specifically, two studies analysed video-taped simulation to evaluate students’ pragmatic competence (Hussin, 2013) and clinical skills (Slingsby et al., 2010); One study used a standardised binary checklist and Integrated Procedural Performance Instrument (IPPI) to assess students’ communication and clinical procedural skills (Nagy et al., 2021), and one incorporated an evaluation criterion with a 4-point scale to compare the quality of case reports written by participants between the real patient group and the standardised patient group (Zhang et al., 2019).

**Table 5:** Key data related to the impacts of SBL and participants’ perceptions

Author (year)	Study aim	Outcomes measured and data collection methods	Key findings relevant to research questions
Adedokun et al. (2022)	To explore the lived experiences of nursing students from CALD backgrounds participating in simulation.	Semi-structured interviews were used to explore students’ lived experiences in simulation.	Eight themes emerged: active participation, strategies for adaptation, mutual connectedness, support from peers and faculty, language challenges, cultural obstacles, vulnerability, and performance pressure.
Hussin (2013)	To investigate how focused reflection on pharmacist-patient simulation can enhance meta-pragmatic awareness and inform pedagogical practices.	Pragmatic competence was measured through analysis of video simulation, semi-structured interviews, and focus group discussions.	Indirectness in student language during pharmacist-patient simulation negatively affected professional communication; guided reflection on simulation performances helped students understand the impact of their language choices; video recordings of students’ simulation sessions were invaluable for identifying areas for improvement.
Rossiter et al. (2023)	To investigate the experiences of domestic and international first-year students engaged in pre-clinical preparation activities, comparing in-person simulation with online simulation.	Pre- and post-surveys using a 5-point Likert scale measured students’ confidence; Focus group was used to explore facilitators and barriers to engagement in simulation.	All students reported an increase in confidence following the simulation, with this effect being particularly more pronounced among international students.
Rutledge et al. (2022)	To investigate the factors that influence medical students’ confidence and perceived competence when performing an FGUE with normal nonpathological findings, focusing on the preference between specialised standardised patients and models, and how student demographics affect these perceptions.	Pre-post surveys with a 5-point Likert scale were used to measure student confidence and perceived competence.	Both first and second-year students, especially those from racial and ethnic minorities, reported higher confidence and perceived competence compared to their local counterparts following simulation.
Harvey et al. (2013)	To report the outcomes of the 2011–2012 Preparation for Practice Program in a nursing program.	Students’ perceived confidence in communication skills, enjoyment levels, and the usefulness of the role plays for clinical practice were measured using surveys combining a 5-point Likert scale statement and open-ended questions.	Students from CALD backgrounds showed increased confidence and high success rates in their clinical practice units following simulation. .
Slingsby et al. (2010)	To explore how Japanese medical students learn clinical reasoning at a US-based workshop.	Linguistic and cultural adaptation, understanding of clinical reasoning, and confidence and communication skills were measured through semi-structured interviews with students, analysis of videotaped standardised patients encounters, and direct observation and field notes.	Structured simulation workshops enhanced students’ understanding of clinical reasoning, improved their communication skills, and increased their confidence by helping them overcome linguistic and cultural barriers.
Zhang et al. (2019)	To improve foreign medical students’ communication skills and their competency in history-taking within a safe learning environment.	The quality of case reports was measured using 4-point scale evaluation criteria containing forty-five items. Students’ perceived difficulties in history-taking, attitudes, and satisfaction with teaching methods were measured through a questionnaire survey.	Incorporating standardised patients significantly improved the quality of medical history-taking case reports and was perceived as an effective method for enhancing communication skills among foreign medical students.
King et al. (2017)	To investigate how effectively standardised patient interactions contribute to academic success among nursing students from CALD backgrounds.	Students’ perceptions of standardised patients were explored through focus groups.	Standardised patients enhanced students’ psychological safety, communication skills, psychomotor abilities, language proficiency, and attitudes toward patient care, while reflective debriefing was essential for consolidating these improvements.
Nagy et al. (2021)	To explore the impact of training communication skills while performing procedural tasks among international and local medical students.	The binary checklist and the IPPI were used to evaluate communication and clinical procedural skills.	Both international and local medical students improved their communication and procedural skills significantly after training with standardised patients, with international students showing a greater relative improvement in communication skills.
Graham & Atz (2015)	To examine baccalaureate minority nursing students’ perceptions of high-fidelity simulation.	Focus group was used to explore the perceptions of students participating in high-fidelity simulation.	Students experienced significant challenges in high-fidelity simulation, including a need to keep the peace, pressure to perform, and issues related to demographics such as isolation and discrimination.

## Themes identified

Overall, three themes were generated from the integration of both qualitative and quantitative data in relation to the impacts of SBL on the learning outcomes of students from CALD backgrounds and their perceptions of participation in SBL activities: (1) diverse learning outcomes of SBL; (2) facing linguistic and cultural challenges that are inherent to SBL; and (3) preparation, reflection, and support to actively participate in SBL activities.

### Theme 1: Diverse learning outcomes of SBL

Nine of the ten included studies identified a variety of learning outcomes associated with SBL. Students from CALD backgrounds in these studies described the benefits of participating in SBL, including increased communication, professional knowledge, clinical skills, confidence, and cultural competence (Graham & Atz, 2015; Harvey et al., 2013; Hussin, 2013; King et al., 2017; Nagy et al., 2021; Slingsby et al., 2010; Rossiter et al., 2023; Rutledge et al., 2022; Zhang et al., 2019).

Students described that through SBL, they learned ways to overcome language barriers by phrasing and conveying complex medical terminology or instructions to patients and families in a simplified and efficient way (King et al., 2017; Rossiter et al., 2023). Medical students reported improved competence in performing a health assessment (King et al., 2017), and enhanced interviewing skills through SBL by practicing the structure and procedure of history-taking (Zhang et al., 2019). SBL also assisted international students in moving beyond their comfort zone. They reported feeling particularly anxious beforehand but noticed a significant increase in confidence after the simulation week, such as being able to speak in front of the group (Rossiter et al., 2023). Following participation in SBL activities, students from CALD backgrounds reported increased cultural competency (King et al., 2017; Slingsby et al., 2010). For example, nursing students overcame stereotypes about older adults, realising that they can lead healthy, productive lives, contrary to their previous beliefs (King et al., 2017). Additionally, female students adjusted to new cultural norms related to gender segregation, feeling more comfortable and at ease when providing care for male patients after interacting with male standardised patients (King et al., 2017).

Overall, students from CALD backgrounds described that SBL provided opportunities for the application of clinical skills and thus improved their sense of preparedness for placements (Harvey et al., 2013; King et al., 2017; Rossiter et al., 2023). Students expressed a sense of psychological safety when interacting with standardised patients in a low-risk environment, which allowed them to practice and refine their skills without fear of negative consequences (King et al., 2017). Students also gained a better understanding of clinical placement expectations; for example, *‘‘When you have dealt with the SPs you know what’s gonna be expected from you*,* like the way you introduce yourself. It like trains [you] to go into the hospital setting’’* (King et al., 2017, p. 526). Similarly, in the study by Harvey et al. (2013), surveyed nursing students from CALD backgrounds reported that role-play in simulation prepared them for the real-life interactions faced in clinical placements.

Three studies compared the learning outcomes of simulation between students from CALD backgrounds and local students (Nagy et al., 2021; Rossiter et al., 2023; Rutledge et al., 2022). Rossiter et al. (2023) found that both international and domestic allied health students reported improved confidence from simulation. However, international students showed a significantly greater increase in confidence regarding foundational clinical skills. Rutledge et al. (2022) reported that medical students from CALD backgrounds had higher confidence and perceived competence in performing female genitourinary exams after SBL than domestic students did. Similarly, Nagy et al. (2021) found that international medical students demonstrated greater improvement in communication performance during clinical procedures, as measured by the Binary Checklist score, following simulation training than their domestic counterparts.

### Theme 2: Facing linguistic and cultural challenges that are inherent to SBL

A range of additional barriers inherent to SBL participation were identified by health professions students from CALD backgrounds, with linguistic and cultural barriers being the most frequently mentioned challenges (Adedokun et al., 2022; Graham & Atz, 2015; Rossiter et al., 2023; Slingsby et al., 2010; Zhang et al., 2019). For example, students described struggles with medical terminologies, unfamiliarity with the local accents, and differences between American and British English (Adedokun et al., 2022); *“you have to translate it… so that makes it [learning] slower for you than for others”*(Adedokun et al., 2022, p. 153). One student described that processing information in a foreign language was particularly effortful, as “*my brain is so tired to process so many English”* (Rossiter et al., 2023, p. 7).

Cultural challenges impacted learning outcomes in SBL in various ways (Adedokun et al., 2022; Slingsby et al., 2010). One nursing student described *“in my culture*,* females are not allowed to do anything [provide care] with the opposite sex. Sometimes when I have to perform care on a male patient*,* a male manikin*,* I feel some kind of weird*”(Adedokun et al., 2022, p. 153). One student perceived some behaviours of other students as uncivil and disrespectful, viewing it as a barrier to their interactions with others, describing as *“adamant*,* sometimes the way they address professors*,* speak to other classmates*,* this is something that back home would definitely not fly by”* (Adedokun et al., 2022, p. 153). In the study by Graham and Atz (2015), nursing students from CALD backgrounds described a lack of cultural diversity in SBL. For example, the exclusive use of white manikins and scenarios focused on white patients did not accurately simulate assessment, diagnosis and treatment in patients of colour, such as detecting pallor or cyanosis (Graham & Atz, 2015). Furthermore, students reported that the use of white manikins heightened their sense of underrepresentation during SBL experiences (Graham & Atz, 2015). In addition, due to different accents and ethnocultural backgrounds, students from CALD backgrounds faced challenges in connecting with native English-speaking peers and experienced socio-cultural isolation. One student described, “*I don’t talk a lot while I’m in the simulation group or even in the clinical group because I know I’m going to mess up my grammar”* (Adedokun et al., 2022, p. 153). Students also experienced discrimination and perceived inferiority from their local peers as underperformers, as one student noted, *“the white students already think I do not know anything”* (Graham & Atz, 2015, p. 485). As a result, these pressures further hindered experiential learning, as students were less engaged in simulation (Adedokun et al., 2022; Graham & Atz, 2015).

### Theme 3: Preparation, reflection, and support to actively participate in SBL activities

Under this theme, a range of factors were identified by students to actively support their participation in SBL activities. Adequate preparation including orientation, pre-reading, introductory content, and goal-setting activities before simulation was viewed by students from CALD backgrounds as beneficial to their learning as this helped them understand the context, set clear objectives, and feel more confident before entering the SBL activities (Adedokun et al., 2022; Rossiter et al., 2023). One student stated: “*looking at the stuff before a class*,* or before the simulation actually help because it makes you have an idea of what you’re going to be doing in the lab”* (Adedokun et al., 2022, p. 152). Students from CALD backgrounds expressed that they required more time to understand concepts of simulation scenarios compared to their native English-speaking peers, so adequate preparation was beneficial and made a significant difference (Adedokun et al., 2022).

Reflective debriefing in SBL activities was viewed by students as an opportunity to understand their limitations and strengths, and to identify areas needing improvement (Adedokun et al., 2022; King et al., 2017; Rossiter et al., 2023). One of the allied students described *“the clinical educator’s feedback would be more convincing like I would trust their feedback and I would learn from their feedback”* (Rossiter et al., 2023, p. 7). Nursing students from CALD backgrounds also emphasised the need for feedback on their nursing care from the viewpoint of standardised patients (King et al., 2017). Some students described that not being able to debrief immediately with simulation facilitators caused significant uncertainty about the quality of their nursing care, which further hindered their engagement in SBL experiences (King et al., 2017). Additionally, students found it difficult to incorporate constructive feedback received from debriefing sessions into practice due to insufficient interaction time with standardised patients, so they expressed a desire for repeated practice within the simulation to master their nursing care skills. (King et al., 2017).

Students from CALD backgrounds reported that receiving support from fellow students from CALD backgrounds during simulation-based activities increased their sense of solidarity, encouragement, connection, and belonging (Adedokun et al., 2022; Rossiter et al., 2023; Slingsby et al., 2010). This peer support was also viewed as helping them overcome barriers related to language and culture (Slingsby et al., 2010). For instance, international students may initially find turn-taking uncomfortable but could grow more comfortable through observation and peer support, thus increasing their confidence and willingness to engage actively in SBL activities (Rossiter et al., 2023). Faculty support was another important factor that students perceived as enhancing their learning and performance (Adedokun et al., 2022; Rossiter et al., 2023). Students in the study by Rossiter et al. (2023) pointed out that the simulation facilitators’ encouragement and confirmation that simulation is a risk-free environment could maintain their engagement in SBL activities. According to Adedokun et al. (2022), nursing students also expressed a preference for working with faculty staff from minority backgrounds during SBL activities, perceiving that the faculty were more understanding, approachable, and willing to help. A student described the perception as “*in terms of language we understand them better than other faculty members and then they consider we ESL students*,* so they after lab stay back to explain things in detail to us*” (Adedokun et al., 2022, p. 153). 

## Discussion

This scoping review aimed to understand how SBL impacts the learning outcomes of health professions students from CALD backgrounds and how students from CALD backgrounds perceive their SBL experiences.

The findings have highlighted the diverse benefits that participation in SBL activities can offer students from CALD backgrounds (Graham & Atz, 2015; Harvey et al., 2013; Hussin, 2013; King et al., 2017; Nagy et al., 2021; Slingsby et al., 2010; Rossiter et al., 2023; Rutledge et al., 2022; Zhang et al., 2019). These benefits align with previous research advocating for tailored educational strategies to support students from CALD, who may face additional barriers in healthcare settings (O’Reilly & Milner, 2015). Interestingly, some of the comparative studies included in this scoping review reported that students from CALD backgrounds demonstrated a significantly greater improvement in performance in clinical skills following SBL than their local counterparts did (Nagy et al., 2021; Rossiter et al., 2023; Rutledge et al., 2022). This could be attributed to the effects of SBL on language skills and cultural understanding among these students. However, these potential reasons have not been thoroughly explored in existing literature, warranting a need for further research. Overall, this scoping review has validated that SBL is a valuable tool for enhancing the clinical skills of students from CALD backgrounds. Future research should explore how different approaches or types of SBL can further support students from CALD backgrounds, examining long-term impacts on their professional integration and patient care practices.

The findings from this scoping review have highlighted that the main factors impacting the learning outcomes of SBL for students from CALD backgrounds are multifaceted and complex. For example, linguistic difficulties appeared to be the most identified barrier that impacted learning outcomes for students from CALD backgrounds participating in SBL activities (Adedokun et al., 2022; Hussin, 2013; King et al., 2017; Rossiter et al., 2023; Zhang et al., 2019). It is crucial to recognise, however, that communication skills extend beyond mere language proficiency. For instance, in the study by Adedokun and colleagues (2022), despite achieving proficiency in English, students still faced communication challenges. Similarly, Hussin (2013) identified that students with high levels of grammatical skills could still adopt inappropriate language when interacting with simulated patients. Furthermore, students from CALD backgrounds may also face greater challenges or require more time to understand new terminologies or complex academic concepts within their disciplines, adding an additional layer of linguistic difficulty (Hussin, 2013). Inadequate confidence or a lack of understanding of cultural nuances can also hinder effective communication, despite students’ perceptions of their English proficiency (Rossiter et al., 2023). It is also important to acknowledge that linguistic difficulties may not solely contribute to interactional challenges among students from CALD backgrounds; rather, their interactional behaviours also play a role (Remedios et al., 2008). Some students from CALD backgrounds in this scoping review found turn-taking uncomfortable during SBL activities, which may be linked to the social demands of social interactions (Rossiter et al., 2023). Students from didactic teaching backgrounds may struggle to adapt to the interactive expectations of SBL, as they are often more familiar with social norms focused on listening rather than active participation (Jeong et al., 2011; Newton et al., 2023; Remedios et al., 2008). Therefore, designing SBL activities involving students from CALD backgrounds requires a comprehensive consideration of these interacting factors. It is not enough to evaluate individual factors in isolation; the interplay between those factors must be thoroughly considered to accurately assess and enhance students’ performance in SBL. This holistic approach can provide a more supportive educational environment, ensuring that all contributing factors to students’ performance are addressed.

To maximise the benefits of SBL, well-designed preparation and reflection opportunities are essential (Adedokun et al., 2022; Harvey et al., 2013; King et al., 2017; Rossiter et al., 2023). Students from CALD backgrounds valued pre-simulation preparation and access to resources prior to participation in SBL activities (Adedokun et al., 2022; Graham & Atz’s, 2015; Harvey et al., 2013; Rossiter et al., 2023) as they needed more time to understand the simulation scenarios and reduce feelings of nervousness (Graham & Atz’s, 2015). However, students reported challenges due to the lack of preparation time before the SBL experiences (Graham & Atz’s, 2015). SBL programs should have well-structured preparatory sessions that consider the additional needs of students from CALD backgrounds without overwhelming them, as processing information and expectations in another language can be particularly overwhelming (Rossiter et al., 2023). Future research could explore how students from CALD backgrounds perceive the duration and structure of these simulation preparation sessions to make these sessions more efficient and effective. In addition to preparation, reflection was valued by students from CALD backgrounds as a crucial component of SBL activities, offering opportunities to identify their strengths and weaknesses (Harvey et al., 2013; Hussin et al., 2013; King et al., 2017; Rossiter et al., 2023). Reflective practice has long been essential in health professions education, primarily based on Kolb’s (1984) experiential learning model which emphasises that learning occurs through both undergoing an experience and reflecting on it. Students in the included studies expressed concerns about the insufficient time for reflection and limited opportunities to apply what they had learned in practice to achieve consistent success (Rossiter et al., 2023). Previous research has shown that clinical supervisors face challenges in guiding students from CALD backgrounds who often lack adequate reflective abilities and require more supervision time (Abu-Arab & Parry, 2015; Nicola-Richmond et al., 2017; O’Reilly & Milner, 2015). It is therefore particularly important to provide adequate reflection opportunities in SBL for students from CALD backgrounds. This will not only enhance their SBL experience but also help them develop own reflective skills for future practice. Additionally, considering the language barriers and reflective capabilities of the CALD group, using guided reflection templates (Sandars, 2009) could facilitate their reflection process for SBL experiences. Furthermore, designing SBL activities that incorporate reflections from previous SBL would be beneficial, helping students to repeatedly practice their skills.

The findings from this scoping review have highlighted that faculty and peer support in SBL activities contribute to the success of SBL for students from CALD backgrounds, providing them with a sense of belonging and psychological safety (Adedokun et al., 2022; Harvey et al., 2013; Rossiter et al., 2023). Faculty staff play significant roles in facilitating reflection and encouraging engagement in SBL (King et al., 2017; Rossiter et al., 2023). Minority staff were particularly perceived by students from CALD backgrounds as strong support systems, being more understanding, approachable, and willing to assist (Adedokun et al., 2022). It is thus beneficial that SBL activities involve staff or simulated patients from diverse ethnic backgrounds as additional supporting resources, although more research in this area is needed. Connecting with peers, especially those from similar backgrounds, can build a support network and promote mutual connectedness due to shared cultural experiences and similar struggles (Adedokun et al., 2022). This finding aligns with a previous scoping review by Metzger et al. (2020), which revealed that perceived cultural similarities fostered strong solidarity among students from CALD backgrounds. Additionally, students from CALD backgrounds demonstrated improved confidence and performance when working with peers of similar ethnic backgrounds, as they could learn from each other (Rossiter et al., 2023). However, some students from CALD backgrounds experienced difficulties connecting and relating well with local students in SBL activities (Adedokun et al., 2022). This finding aligns with those of previous studies that highlighted the challenges students from CALD backgrounds face in interacting with domestic students in both academic and social contexts (Gilligan & Outram, 2012; Lim et al., 2016). This could be explained by linguistic difficulties and fewer shared interests with domestic students, and a home culture that emphasises an intense focus on marks as well as listening quietly and attentively (Lim et al., 2016). In addition, students from CALD backgrounds in SBL activities faced fault-finding attitudes from domestic peers, who perceived them as less intelligent and underperformers (Adedokun et al., 2022; Graham & Atz’s, 2015). Such a focus on weaknesses and low-performance expectations placed pressure on those students during SBL activities, which impaired their active participation (Adedokun et al., 2022; Graham & Atz’s, 2015). The feeling of isolation, along with instances of rejection and discrimination from domestic students and faculty, has been noted in SBL (Adedokun et al., 2022; Graham & Atz’s, 2015) and university general group activities from previous research (Jeong et al., 2011). To design inclusive SBL activities, simulation facilitators should consider how students are allocated to ensure that students from CALD backgrounds receive support from fellow CALD peers while also creating adequate opportunities for interaction with domestic students. Future research should also explore in-depth the attitudes and stereotypes that students from CALD backgrounds, domestic students, and staff hold towards each other when participating in SBL activities. This mutual understanding could inform strategies to foster more positive interactions among all parties involved.

## Future directions

Most studies in this scoping review were concentrated within the fields of nursing and medicine, which is consistent with previous research concerning students from CALD backgrounds in health professions programs. It is important to acknowledge that each health profession has additional clinical preparation needs, thus differing needs for specific clinical skills and the use of SBL to achieve different objectives. Therefore, future research should explore SBL for students from CALD backgrounds across diverse health professions programs.

It has been noted that both ‘simulated patients’ and ‘standardised patients’ were used across studies, but there was a lack of clarity regarding these terms. Clear definitions of the differences between these concepts are very important as they differ and require distinct structures in their preparation, development, and delivery (Churchouse & McCafferty, 2012). This scoping review remained faithful to the original use of those two terms to avoid confusion. However, future research should provide a clear definition of the type of SBL used.

Most studies in this scoping review did not provide detailed descriptions of the steps and durations of SBL, which hinders replicability, leads to inconsistent findings, and limits generalisability. Without these details, it is difficult to understand which aspects of SBL are most beneficial. Future research should aim to develop standardised reporting guidelines to include comprehensive protocols detailing the steps and durations of SBL. Future research could also explore different structures and durations of SBL sessions to identify the most effective formats for enhancing clinical skills among students from CALD backgrounds.

None of the studies in this scoping review explored how students from CALD backgrounds contribute to the SBL learning environment, despite existing literature highlighting the benefits of their inclusion in health professions programs. Future research should consider a strengths-based approach to explore the contributions that these students make to SBL.

## Scoping review limitations

The limitations of this scoping review are acknowledged. Firstly, the topic of students from CALD in SBL is relatively new, with only a limited body of research available and a lack of recent literature. Secondly, as Pham et al. (2021) have described, CALD is a complicated concept without a standardised definition for CALD used within the literature. To address this issue, this scoping review employed broad terms relevant to CALD to search the databases. Consequently, the research team identified and reviewed a larger number of articles. However, the lack of consistent terminology could have caused some relevant studies to be missed in the literature review. Thirdly, the decision to include only studies with primary aims relevant to SBL and students from CALD backgrounds may have led to the omission of some results generated within broader educational programs or populations that may be relevant to these discussions. However, this approach ensured that the results included in this scoping review were highly relevant. Finally, inherent to the nature of scoping reviews, this study did not aim to critique the quality of the included articles but focused on the breadth of evidence (Munn et al., 2018). However, while this scoping review included peer-reviewed empirical studies and excluded grey literature, this approach may limit the comprehensiveness of the review by potentially missing unpublished insights. Nonetheless, it ensures a focus on verified research findings. Despite these limitations, this review has yielded important findings that can guide future work in this emerging field.

## Conclusion

This scoping review has outlined evidence of the benefits of SBL for health professions students from CALD backgrounds. The importance of tailored SBL strategies that address specific learning needs among these students has been highlighted. As the field continues to evolve, it is crucial that future research builds on these findings and further explores and refines SBL practices to support the diverse needs of all students in health professions programs. Further research in this space will contribute to the development of more inclusive and effective educational frameworks in health professions education.

## Data Availability

No datasets were generated or analysed during the current study.
